# Teneligliptin alleviates diabetes-related cognitive impairment by inhibiting the endoplasmic reticulum (ER) stress and NLRP3 inflammasome in mice

**DOI:** 10.18632/aging.205333

**Published:** 2023-12-19

**Authors:** Weifeng Wang, Juanjuan Zhang

**Affiliations:** 1 Department of Endocrinology, Laizhou City People’s Hospital, Yantai, Shandong 261400, China

**Keywords:** teneligliptin, diabetes-related cognitive impairment, NLRP3, ER stress

## Abstract

Diabetes mellitus (DM) significantly influences the normal health of patients with its severe complications, including diabetes-related cognitive impairment (CI). Recently, neuroinflammation and oxidative stress (OS) have been reported to participate in the pathogenesis of diabetes-related CI. Teneligliptin, an inhibitor of DDP-IV, was developed for treating DM and is claimed with promising effects against inflammation. Herein, in the current study, we examined the potential therapeutic function of Teneligliptin against diabetes-related CI. Db/m or diabetic mice were orally administered with teneligliptin (60 mg/kg/day) for 10 weeks. Elevated levels of total cholesterol (TC), triglyceride (TG), and low-density lipoprotein cholesterol (LDL-C), increased escape latency, declined time in the platform quadrant and decreased number of platform crossings in the Morris water maze test, reduced freezing index in the fear conditioning test, and lessened time spent in the novel arm and percentage of alterations in the Y-maze test were observed in diabetic mice, all of which were sharply improved by teneligliptin. Furthermore, increased levels of inflammatory cytokines and activated OS state were observed in the hippocampus of diabetic mice, which were markedly repressed by Teneligliptin. Lastly, the activation of the NOD-like receptor family pyrin domain containing 3 (NLRP3) signaling and the endoplasmic reticulum (ER) stress pathway in the hippocampus of diabetic mice were notably inhibited by teneligliptin. Collectively, teneligliptin mitigated diabetes-related CI by repressing the ER stress and NLRP3 inflammasome in diabetic mice.

## INTRODUCTION

Diabetes mellitus (DM) is a serious metabolic disease with increasing complications and mortality associated with DM. According to epidemiological surveys, nearly 400 million diabetes patients are diagnosed annually in the world, of which 90–95% are type 2 diabetes mellitus (T2DM) [1]. It is expected that by 2035, the number of worldwide diabetic patients will reach 600 million [2]. Multiple serious complications are reported on T2DM, including CI [3]. Therefore, it is urgent to seek methods for treating DM complications. Significant cognitive decline is reportedly observed in DM patients, about 70% of which eventually develop into Alzheimer’s disease (AD). A much higher risk of AD is reported in DM patients [4, 5]. CI refers to the early stage of dementia, in which patients show cognitive dysfunction at the time of testing while retaining their basic ability to live [6]. Compared to subjects with normal blood glucose, the risk of CI in patients with diabetes is 0.5- times higher and the risk of dementia is 1- time higher [7]. Diabetes-related CI has a negative impact on learning ability, memory, attention, and executive ability, and even causes emotional disorders such as depression and anxiety [8, 9]. Inflammatory response is one of the important causes of CI in diabetes. Long-term hyperglycemia is a major feature of diabetes, which induces the inflammatory response by activating the NLRP3 inflammasome to promote the production of interleukin (IL)-1β [10]. Furthermore, the glycolysis intermediates are accumulated by persistent hyperglycemia to induce the accumulation of advanced glycation end products (AGEs), which further activate the NF-κB pathway to induce the secretion of a variety of inflammatory factors by microglia. By binding to the RAGE, AGEs induce the release of reactive oxygen species (ROS) to activate NF-κB signaling, which finally contributes to the excessive production of inflammatory factors [11, 12]. Therefore, controlling neuroinflammatory response may become an important direction for treating diabetes-related CI.

As an inhibitor of DDP-IV, teneligliptin is a novel antidiabetic drug that inhibits the degradation of glucagon-like peptide 1 (GLP-1) via repressing the activity of DDP-IV. It exerts its antidiabetic function by increasing the blood concentration of GLP-1 [13, 14]. Teneligliptin was first developed by Mitsubishi Tanabe Pharma and approved for treating T2DM in Japan in 2012, with its promising antidiabetic function proven in several clinical trials [15, 16]. DPP-4 inhibitors could be classified into peptidomimetic (i.e., sitagliptin, vildagliptin, saxagliptin, and anagliptin) and non-peptidomimetic (i.e., alogliptin and linagliptin) subtypes. Teneligliptin has a different structure and pharmacodynamic characteristics from other gliptins. These features could confer properties diverse from or additive to other DPP-4 inhibitors. Firstly, it is a potent, selective, and long-lasting inhibitor of DPP-4, and exhibits strong inhibitory activity via its J-shaped structure and ‘anchor lock domain’. Secondly, teneligliptin has high tissue distribution [17]. An X-ray co-crystal structure of teneligliptin with DPP-4 demonstrates that the key interaction occurs between the phenyl ring on the pyrazole and the S2 extensive subsite of DPP-4, which not only enhances the potency of the drug but also increases its selectivity [18]. Recently, several researches have claimed that teneligliptin shows prominent inhibitory effects against inflammation [19–21]. Also, teneligliptin was found to ameliorate high glucose-induced ER stress in endothelial cells [22]. However, the function of teneligliptin in diabetes-related CI remains uncertain. Herein, the preliminary investigation of teneligliptin against diabetes-related CI was conducted in db/db mice.

## MATERIALS AND METHODS

### Animals and grouping

12 non-diabetic (db/m) and 12 db/db mice (7–9 weeks) were obtained from the Vital River (China). After one week of adaptive feeding, db/m mice and db/db mice were orally administered with teneligliptin (60 mg/kg/day) for 10 weeks. The mice were divided into four groups: db/m, db/m+ teneligliptin, db/db, and db/db+ teneligliptin.

### The detection of serum levels of TC, TG, and LDL-C

The peripheral blood was collected to obtain the serum, followed by detecting the TC, TG, and LDL-C levels in the serum using the fully automatic biochemical analyzer (HITACHI, Japan).

### Morris water maze test

The black maze was divided into 4 quadrants and the platform was placed in the 3rd quadrant. Water was poured into the maze to cover the platform, and differentiated by shapes and colors. The training was initiated by putting the mouse’s head toward the wall of the pool in the other 3 quadrants however it halted- when the mouse climbed to the platform. The escape latency was recorded as the time the mouse took to find the hidden underwater platform. On the 6th day of training, the mouse was put in the maze to test its spatial memory. The mouse was submerged in water for 60 s, and its spatial memory was assessed based on the time spent in the platform quadrant and the number of platform crossings.

### Fear conditioning test

The experiment was divided into two stages: conditioned fear experimental training and situational conditioned fear experimental testing. The activity trajectory of the mouse was automatically tracked using the image automatic monitoring system (XR-XC404, Xinruan Information, China). The conditioned fear test was performed on the second day after the operation. Mice were placed in a sound-proof training box for 180 s, after which they received sound (30 s, 75 dB, 3 000 Hz) and plantar shock (2 s, 0.75 mA, and the last 2 s of shock before the end of the sound). 24 h after training, the situational fear conditioning test was performed. Mice were put back into the original experimental chamber without any stimulation, and the time of freezing (an inactive state with no other behavior except breathing) within 5 min was recorded.

### Y-maze test

The equipment consisted of three identical arms (40 cm × 8.5 cm × 15 cm, angle 120°), designated as A, B, and C, respectively. Each mouse was put in the middle area of the maze at the beginning of the experiment. Subsequently, the mouse was allowed to probe freely for 8 mins, and the Noldus movement trajectory tracking system was used to record the time spent in the novel arm and the number of correct rotations (continuously entering 3 different arms, such as ABC, BCA, etc.). The percentage of alterations was calculated as (number of rotations/maximum number of rotations -2) ×100.

### Enzyme-linked immunosorbent assay (ELISA) for the cytokine level and glutathione peroxidase (GSH-PX) activity detection

The mice were sacrificed in a sealed container collected with a CO_2_ tanker. The hippocampus of each mouse was collected and homogenized, followed by centrifugation and collection of the supernatant. 50 μL supernatant was diluted at a 1:1 ratio and loaded into the well. Then, 50 μL Biotin-labeled antibody was introduced and cultured for 60 min at 37°C, followed by removing the reagent and adding 80 μL horseradish peroxidase (HRP)-loaded secondary antibody. Following half an hour of culturing at 37°C, 50 μL TMB substrates were added and cultured at 37°C for 10 min, followed by loading 50 μL stop solution. Lastly, the optical density (OD) value was achieved using a microplate reader (MD, USA).

### The measurement of malondialdehyde (MDA) level, Superoxide dismutase (SOD) activity in the hippocampus

The hippocampus was collected and homogenized, followed by centrifugation and collecting the supernatant. The MDA level (Jiancheng, China) and SOD activity (Solarbio, China) in the hippocampus were determined with a commercial kit using the TBA and WST-1 methods, respectively. The instructions were strictly followed.

### Real-time polymerase chain reaction (RT-PCR) assay

Total RNAs were obtained from the hippocampus using the TRIzol reagent and were quantified with the ultraviolet spectrophotometer (Hach, USA). A cDNA synthesis kit (SolelyBio, China) was utilized for conducting the transcription from RNAs to cDNAs, followed by performing the PCR reaction utilizing an SYBR Premix Ex TaqII kit (Takara, Japan). The 2^−ΔΔCt^ method was used for the calculation of gene levels. The primer sequences for the genes used in this experiment are listed as follows: IL-1β FW5′-TTCGA GGCAC AAGGC ACAA-3′; RV5′-CCATC ATTTC ACTGG CGAGC-3′; MCP-1 FW5′-GCTCA GCCAG ATGCA AT-3′; RV5′-GCTTG TCCAG GTGGT CCATG-3′; IL-6 FW5′-GACAA AGCCA GAGTC CTTCA GAGAG ATACA G-3′; RV5′-TTGGA TGGTC TTGGT CCTTA GCCAC-3′; β-actin-FW5′-CATGT ACGTT GCTAT CCAG GC-3′; RV5′-CTCCT TAATG TCACG CACGA T-3′.

### Western blot analysis

The hippocampus was collected and lysed to obtain total proteins and quantified with the BCA method. After separation using the sodium dodecyl sulfate (SDS)-polyacrylamide gel (PAGE), proteins were delivered to the polyvinylidene fluoride (PVDF) membrane, followed by blocking in 5% skim milk. Primary antibodies against thioredoxin-interacting protein (TXNIP) (1:2000, Cat#abs136992, Absin, China), apoptosis-associated speck-like protein (ASC) (1:1000, Cat#abs155599, Absin, China), NLRP3 (1:2500, Cat#abs151715, Absin, China), C/EBP Homologous Protein (CHOP) (1:1000, Cat#abs131376, Absin, China), phosphorylated-PERK (1:1000, Cat#abs137056, Absin, China), PERK (1:1000, Cat#abs124201, Absin, China), phosphorylated inositol-requiring enzyme 1alpha (p-IRE1α) (1:1000, abs127778, Absin, China), IRE1α (1:1000, abs127778, Absin, China), ATF6 (1:2000, #65880, CST, USA), or β-actin (1:2000, Cat#8457CST, USA), the HRP-linked, Anti-rabbit IgG (1:2000, Cat#7074, CST, USA) were introduced and cultured at 4°C overnight. Subsequently, the HRP-linked, anti-rabbit IgG (1:2000, Cat#7074, CST, USA) was loaded and cultured for 90 min at 37°C. Lastly, ECL solution was utilized for the exposure of the bands and the ImageJ software was used for expression level analysis.

### Statistical analysis

Data were listed as mean ± Standard Deviation (SD), which were analyzed using the one-way analysis of variance (ANOVA) method by Tukey’s test with the software SPSS (Version 24.0). *P* < 0.05 was considered statistically significant.

### Data availability

The data is available upon reasonable request from the corresponding author.

## RESULTS

### Teneligliptin alleviated dyslipidemia in diabetic mice

**Figure 1:**
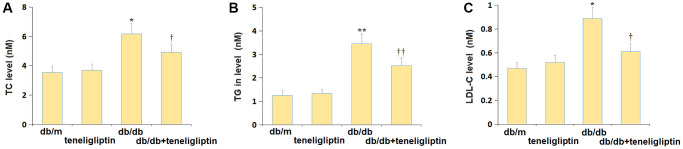
**Teneligliptin improved the dyslipidemia in db/db mice.** (**A**) Total cholesterol (TC) level; (**B**) Triglyceride (TG) in level; (**C**) Low-density lipoprotein cholesterol (LDL-C) level (^*, **^P < 0.05, 0.01 vs. db/db mice group; ^†, ††^*P* < 0.05, 0.01 vs. Teneligliptin group).

Firstly, the biochemical indexes were detected following administration. The TC level was maintained around 3.6 mM in the db/m and db/m+ teneligliptin groups and was signally increased to 6.18 mM in the db/db group, then markedly reduced to 4.92 mM by teneligliptin (Figure 1A). Furthermore, the TG levels in the db/m, db/m+ teneligliptin, db/db, and db/db+ teneligliptin groups were 1.25, 1.33, 3.46, and 2.52 mM, respectively (Figure 1B). Moreover, the LDL-C level was slightly altered in the db/m+ teneligliptin group and notably elevated in the db/db group but was dramatically declined in the db/db+ teneligliptin group (Figure 1C).

### Teneligliptin alleviated the behavioral dysfunction of diabetic mice in the Morris water maze test

**Figure 2:**
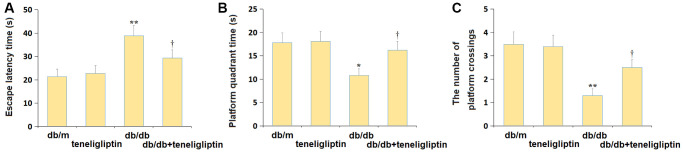
**Teneligliptin alleviated the behavioral dysfunction of db/db mice in the Morris water maze test.** (**A**) The escape latency to reach the escape platform; (**B**) The time spent in the platform quadrant; (**C**) The number of platform crossings (^*, **^P < 0.05, 0.01 vs. db/db mice group; ^†, ††^*P* < 0.05, 0.01 vs. Teneligliptin group).

The escape latency to reach the escape platform in the db/m, db/m+ teneligliptin, db/db, and db/db+ teneligliptin groups was 21.4, 22.7, 38.9, and 29.3 s, respectively (Figure 2A). The time spent in the platform (Figure 2B) was kept around 18.0 s in the db/m and db/m+ teneligliptin groups, and was markedly reduced to 10.8 s in diabetic mice, then signally increased to 16.2 s by teneligliptin. Furthermore, the average number of platform crossings in the db/m, db/m+ teneligliptin, db/db, and db/db+ teneligliptin groups was 3.5, 3.4, 1.3, and 2.5, respectively (Figure 2C).

### Teneligliptin mitigated the behavioral dysfunction of diabetic mice in the fear conditioning test

**Figure 3:**
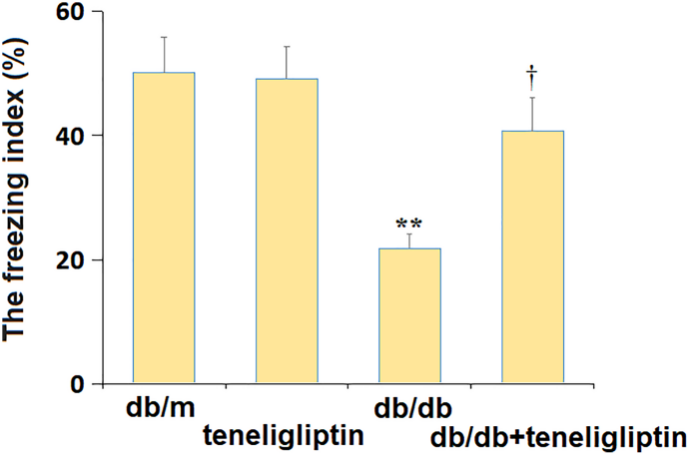
**Teneligliptin mitigated the behavioral dysfunction of db/db mice in the fear conditioning test.** The freezing index (%) (^*, **^P < 0.05, 0.01 vs. db/db mice group; ^†^*P* < 0.05 vs. Teneligliptin group).

Subsequently, the behavior change in diabetic mice was confirmed using the fear conditioning test. The freezing index was minorly changed from 50.1% to 49.2% in the db/m+ teneligliptin group and largely decreased to 21.8% in the db/db group, then greatly reversed to 40.7% by teneligliptin (Figure 3).

### Teneligliptin ameliorated the behavioral dysfunction of diabetic mice in the Y-maze test

**Figure 4:**
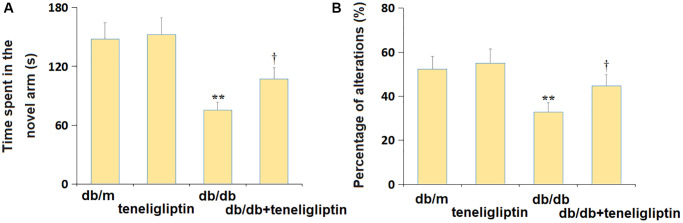
**Teneligliptin ameliorated the behavioral dysfunction of db/db mice in the Y-maze test.** (**A**) Time spent in the novel arm; (**B**) Percentage of alterations (%) (^*, **^P < 0.05, 0.01 vs. db/db mice group; ^†^*P* < 0.05 vs. Teneligliptin group).

In the Y-maze test, the time spent in the new arm was minorly changed from 148.2 s to 152.4 s, largely declined to 75.4 s in diabetic mice, then dramatically increased to 107.2 s by teneligliptin (Figure 4A). Moreover, the percentage of alterations in the db/m, db/m+ teneligliptin, db/db, and db/db+ teneligliptin groups was 52.3%, 55.1%, 32.9%, and 44.7%, respectively (Figure 4B).

### Teneligliptin repressed the production of inflammatory cytokines in diabetic mice

**Figure 5:**
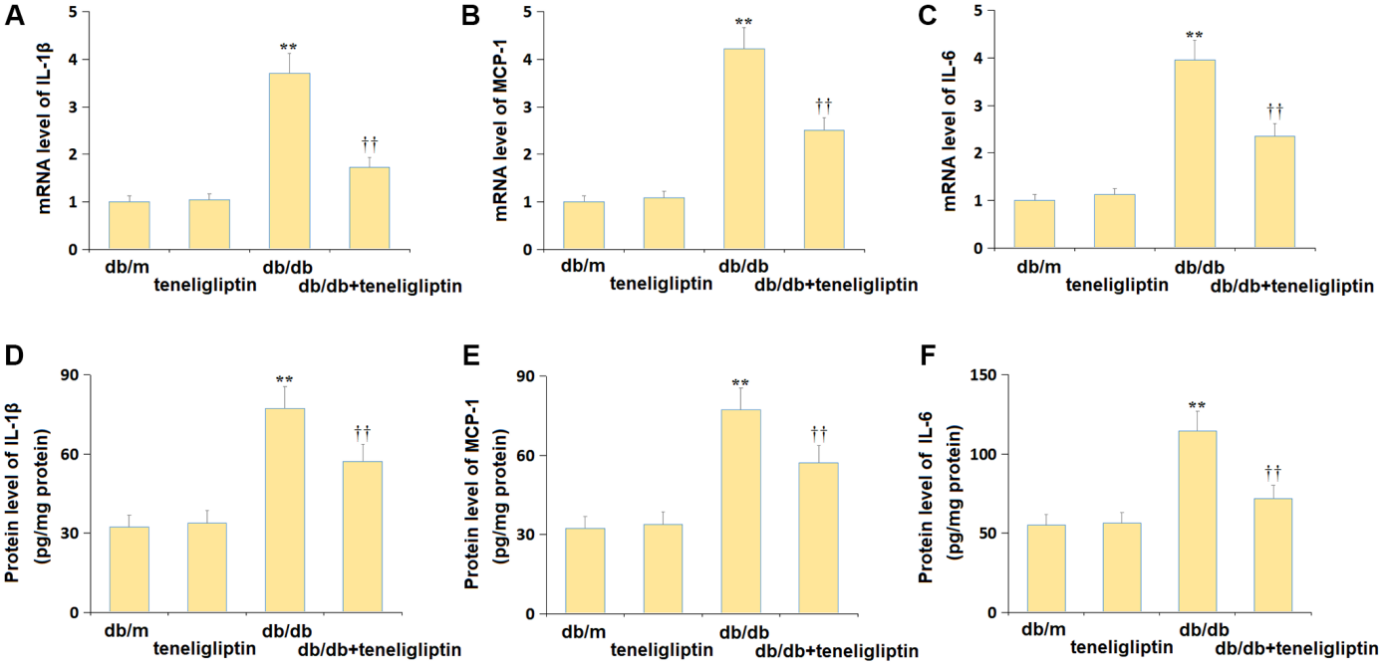
**Teneligliptin repressed the production of inflammatory cytokines in the hippocampus of db/db mice.** (**A**) mRNA level of IL-1β, (**B**) mRNA level of MCP-1, and (**C**) mRNA level of IL-6; (**D**) Protein level of IL-1β, (**E**) Protein level of MCP-1, and (**F**) Protein level of IL-6 (^**^P < 0.01 vs. db/db mice group; ^††^0.01 vs. Teneligliptin group).

The state of neuroinflammation in the hippocampus was investigated. The gene levels of IL-1β, MCP-1, and IL-6 were mildly changed in the db/m+ teneligliptin group, markedly elevated in the db/db group, then memorably reduced in the db/db+ teneligliptin group (Figure 5A–5C). The IL-1β levels in the db/m, db/m+ teneligliptin, db/db, and db/db+ teneligliptin groups were 32.4, 33.8, 77.4, and 57.3 pg/mg protein, respectively (Figure 5D). The MCP-1 level was kept around 400.0 ng/mg protein in the db/m and db/m+ teneligliptin groups was markedly increased to 702.5 ng/mg protein in diabetic mice, then greatly decreased to 569.8 ng/mg protein by teneligliptin (Figure 5E). Furthermore, the IL-6 levels in the db/m, db/m+ teneligliptin, db/db, and db/db+ teneligliptin groups were 54.9, 56.3, 114.7, and 71.9 pg/mg protein, respectively (Figure 5F).

### Teneligliptin reversed the OS in diabetic mice

**Figure 6:**
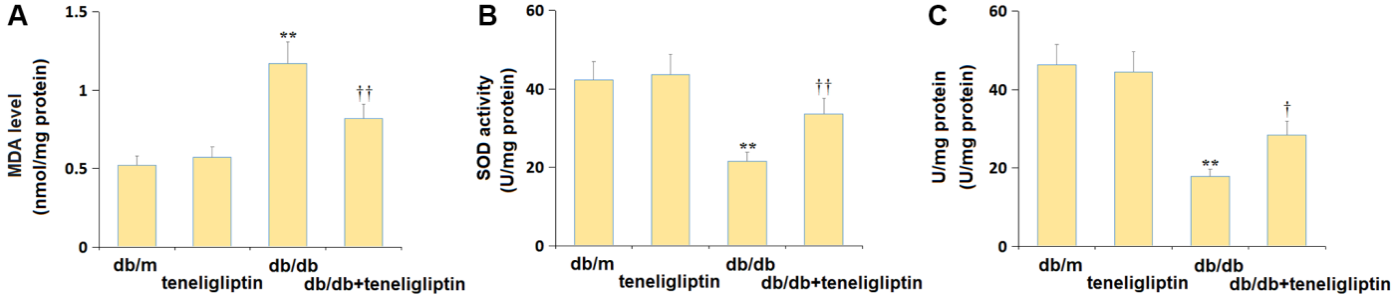
**Teneligliptin reversed the oxidative stress in the hippocampus of db/db mice.** (**A**) MDA level; (**B**) SOD activity; (**C**) GSH-PX activity (^**^P < 0.01 vs. db/db mice group; ^††^0.01 vs. Teneligliptin group).

The OS state in the hippocampus of each mouse was further studied. The increased MDA level in diabetic mice was found markedly repressed by teneligliptin (Figure 6A). The SOD activity in the db/m, db/m+ teneligliptin, db/db, and db/db+ teneligliptin groups was 42.3, 43.7, 21.5, and 33.6 U/mg protein, respectively (Figure 6B). The GSH-PX activity was retained around 45.0 U/mg protein in the db/m and db/m+ teneligliptin groups was notably decreased to 17.8 U/mg protein in diabetic mice, then markedly reversed to 28.4 U/mg protein by teneligliptin (Figure 6C).

### Teneligliptin inhibited the NLRP3 inflammasome in diabetic mice

**Figure 7:**
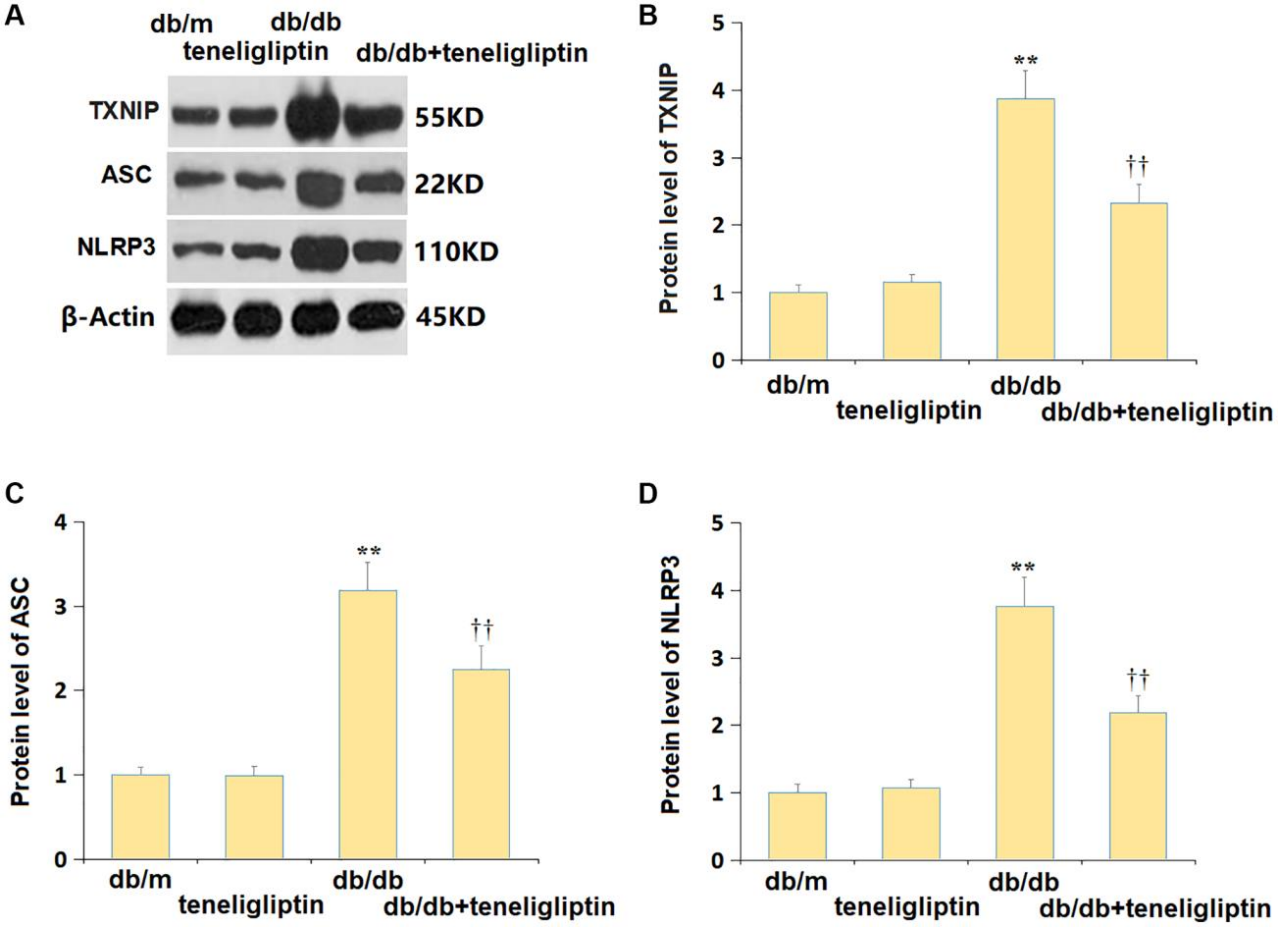
**Teneligliptin inhibited the NLRP3 inflammasome in the hippocampus of db/db mice.** (**A**) Protein level of genes were determined using western blots; (**B**) Protein level of TXNIP; (**C**) Protein level of ASC; (**D**) Protein level of NLRP3 (^**^P < 0.01 vs. db/db mice group; ^††^0.01 vs. Teneligliptin group).

NLRP3 signaling has a claimed correlation to the development of diabetes-related CI [23]. Herein, the mildly altered TXNIP, ASC, and NLRP3 levels in the db/m+ teneligliptin group were markedly promoted in diabetic mice, and then signally repressed by teneligliptin (Figure 7A–7D).

### Teneligliptin alleviated the ER stress in diabetic mice

**Figure 8:**
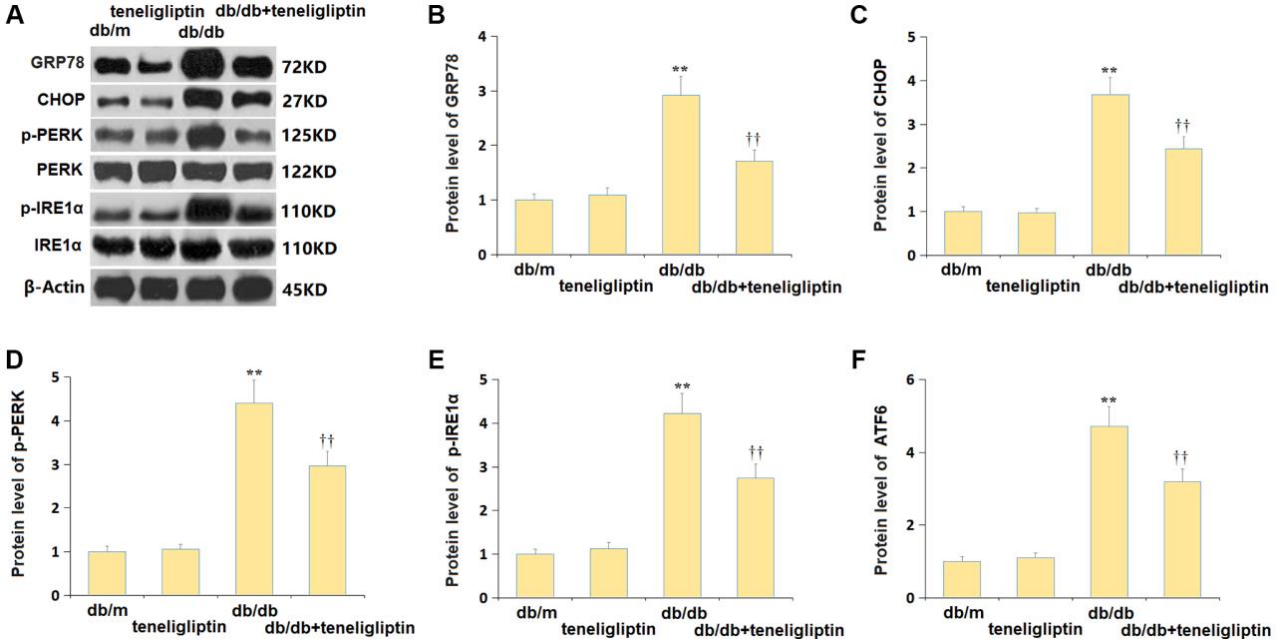
**Teneligliptin alleviated the ER stress in the hippocampus of db/db mice.** (**A**) Protein level of genes were detected by western blots; (**B**) Protein level of GRP78; (**C**) Protein level of CHOP; (**D**) Protein level of p-PERK; (**E**) Protein level of p-IRE1α; (**F**) Protein level of ATF6 (^*, **^P < 0.05, 0.01 vs. db/db mice group; ^†, ††^*P* < 0.05, 0.01 vs. Teneligliptin group).

ER stress is another important mechanism contributing to the pathogenesis of diabetes-related CI [24]. Herein, the levels of ER stress-related proteins, including GRP78, CHOP, p-PERK, p-IRE1α, and ATF6, were minorly altered in the db/m+ teneligliptin group, largely increased in the db/db group, then markedly suppressed in the db/db+ teneligliptin group (Figure 8A–8F).

## DISCUSSION

DPP-4 inhibitors are potent drugs used to treat patients with T2DM [25, 26]. Inhibition of DPP-4 increases levels of biologically intact incretins, improving glucose metabolism through the upregulation of insulin secretion and the suppression of glucagon release [27]. Recently, several articles have reported on its neuroprotective effects in both *in vivo* and *in vitro* models [28, 22]. The inflammatory response is a critical mechanism in DM and diabetes-related CI [29]. Hyperglycemia and ROS are critical factors inducing an inflammatory response in diabetes-related CI patients. When an inflammatory response occurs, microglia in the hippocampus related to learning and memory function release a large number of inflammatory factors, which further contribute to neuronal degeneration [30]. The persistent occurrence of chronic inflammation changes the permeability of the blood-brain barrier (BBB) [31], and a large number of toxic and harmful substances cross the BBB and enter the brain, increasing the burden on the nervous system and causing neurodegenerative diseases. The secretion of pro-inflammatory factor IL-1β is found to induce neuroinflammation, which is the key to the pathogenesis of diabetes-related CI [32]. The levels of inflammatory factors in diabetes-related CI patients are dramatically higher than those in DM patients [29]. Herein, changed biochemical criteria observed in diabetic mice were consistent with data reported previously [33]. Furthermore, in diabetic mice, behavioral dysfunction was observed in the Morris water maze, the fear conditioning, and the Y-maze tests, in line with the research published by Wu [34]. After the administration of teneligliptin, biochemical criteria were markedly improved, accompanied by alleviation of the behavioral dysfunction, implying that teneligliptin exerted a promising protective function on CI in diabetic mice. Moreover, similar to data presented by Shang [35], severe neuroinflammation and activated OS state were observed in diabetic mice, both of which were sharply ameliorated by teneligliptin, suggesting that the protection of teneligliptin against CI in diabetic mice might be correlated to its repression on the inflammation and OS.

The secretion and maturation of IL-1β is regulated by the inflammatory activation of NLRP3, which induces various forms of inflammatory response, thereby promoting the development of central nervous system diseases [36–38]. IL-1β is also an important mediator of accelerating neurodegenerative diseases and diabetes-related CI [39], and significantly increased hippocampal IL-1β levels are associated with cognitive and emotional changes in diabetic mice [40]. Animal studies have shown that diabetic complications are alleviated by the inhibitor of the NLRP3 inflammasome [41, 42]. Herein, high levels of IL-1β were observed in diabetic mice, accompanied by an activation of the NLRP3 inflammasome, consistent with data reported by Tian [43] and Lu [44]. Moreover, the level of IL-1β and the activation of the NLRP3 inflammasome were notably suppressed by teneligliptin, indicating that the function of teneligliptin in diabetic mice was correlated to the inhibition of NLRP3 signaling. In future work, the association between the NLRP3 inflammasome and teneligliptin in diabetes-related CI will be identified by co-administering the diabetic mice with teneligliptin and the agonist of the NLRP3 inflammasome.

ER is an important organelle that participates in protein folding, transport, and maintaining calcium ion balance. When the pressure load of protein folding in the cell is low, Bip/GRP78 in the ER binds to the luminal segment of the ER transmembrane proteins ATF6, IRE-1, and PERK, which is in an inactive state. However, when the folding capacity of ER cannot meet the demand of newly synthesized unfolded protein or the calcium ion balance is broken, cells will be in the ER stress state [45, 46]. ER stress signaling is closely related to various pathological factors and states of cognitive decline in diabetes, such as metabolic abnormalities, cerebral ischemia, insulin resistance, neuronal calcium homeostasis, neurotransmitter changes, inflammatory response, and oxidative stress [47]. ER stress induces neuronal apoptosis through GSK3/3β, C/EBP-homologous protein (CHOP), and Caspase-12 signaling pathways or interacting with the mitochondrial apoptosis pathway [48–51]. Herein, consistent with the research by He [52], the remarkably activated ER stress pathway in diabetic mice was markedly repressed by teneligliptin, implying that the function of teneligliptin in diabetic mice was correlated to the suppression of the ER stress pathway. The involvement of the ER stress pathway in the regulatory mechanism of teneligliptin in diabetes-related CI will be further confirmed by co-administering diabetic mice with teneligliptin and tunicamycin, the activator of ER stress.

In conclusion, teneligliptin alleviated diabetes-related CI by repressing the ER stress and NLRP3 inflammasome in diabetic mice. Further studies can help clarify whether a sufficient improvement in cognitive impairment could result from long-term use of teneligliptin therapy in diabetic patients.
